# Supplementary material for: Risk factors for blood vessel rupture during vascular access intervention therapy for hemodialysis patients
Source: PLoS One. 2023 Mar 31;18(3):e0283844. doi: 10.1371/journal.pone.0283844 (PMC10065258; doi:10.1371/journal.pone.0283844)
Supplement: Attachment — Submitted filename: Response to Reviewers.docx [file pone.0283844.s002.docx]

February 25, 2023

Dear Dr. Tze-Woei Tan

Academic Editor, *PLOS ONE*

We hereby submit a revised version of our manuscript.

(PONE-D-22-35142)

**Authors:** Yui Kambayashi, Ken Iseri, Tomoki Morikawa, Atushi Yao, Akio Yokochi, Hirokazu Honda

**Title:** Risk factors for blood vessel rupture during vascular access intervention therapy for hemodialysis patients

Thank you for inviting us to submit a revised draft of our manuscript to *PLOS ONE*. We appreciate the time and effort you and each of the reviewers have dedicated to providing insightful feedback on ways to strengthen our paper. Thus, it is with great pleasure that we resubmit our article for further consideration. We have incorporated changes that reflect the detailed suggestions you have graciously provided. We also hope that our edits and the responses we provide below satisfactorily address all the issues and concerns you and the reviewers have noted.

To facilitate your review of our revisions, the following is a point-by-point response to the questions and comments. New text added to the manuscript is marked as red in the rebuttal and in the revised manuscript.

**Reviewer #1:**

Reviewer #1: This is a retrospective study analyzing the risk factors for vascular rupture during interventional therapy. This study is trying to answer a very critical clinical question, but the manuscript needs to provide more information
We are grateful for your comments and suggestion to improve our research. We have changed the manuscript according to your suggestions (see added text below).

1. How were the study patients selected? What is the referral criteria for VAIVT?

**RESPONSE:** To address this concern, we have added the following sentences in the manuscript.

“At our hospital, dialysis patients undergo routine ultrasound scans for early detection of stenosis as part of their care. Based on Japanese vascular access guidelines recommending VAIVT if blood quantity flow is under 400ml/min or the resistance index is over 0.6, 211 patients received VAIVT using ultrasound at Kanto Rosai Hospital from November 2019 to December 2021. No patients were excluded. Thus, the analysis was performed on 628 VAIVT procedures performed on 211 patients.”

2. Are patients in Japan received routine ultrasound screening or patients is referred when they have issues with their accesses?

**RESPONSE**: In Japan, the availability of routine ultrasound screening for early detection of stenosis varies among healthcare facilities, but it is believed that most dialysis patients receive it. At our hospital, dialysis patients undergo routine ultrasound scans as part of their care.

3. what is the location of the AVG? how many patients have AVF? Location of the fistula?

**RESPONSE**: We are grateful for your comments. Among a total of 211 patients in the study, 198 patients had AVF. Location of the fistula was listed in below.

|  |  | **Not rupture** | **Rupture** | **P-value** |
| --- | --- | --- | --- | --- |
| **AVF/AVG location** | basilic vein-median cubital vein | 1(0.2%) | 0(0%) | 0.55 |
|  | brachial artery- basilic vein | 21(3.5%) | 0(0%) |  |
|  | brachial artery- brachial vein | 22(3.7%) | 0(0%) |  |
|  | brachial artery- cephalic vein | 9(1.5%) | 0(0%) |  |
|  | ex AVG-brachial artery | 9(1.5%) | 0(0%) |  |
|  | ex AVG-brachial vein | 3(0.5%) | 0(0%) |  |
|  | ex AVG-cephalic vein | 3(0.5%) | 1(5.0%) |  |
|  | elbow fistula | 32(5.4%) | 0(0%) |  |
|  | radiocephalic fistula | 478(80.1%) | 18(90.0%) |  |
|  | AVF in tabaciere region | 1(0.2%) | 0(0%) |  |
|  | ulnobasilic fistula | 18(3.0%) | 1(5.0%) |  |

4. What is the average vintage of dialysis access?

**RESPONSE:** Thank you for meaningful comment. we have added data in the table.

|  |  | **Not rupture** | **Rupture** | **P-value** |
| --- | --- | --- | --- | --- |
| **Access vintage(year)** |  | 2(1-5) | 1(1-3) | 0.017 |

5. What is the rupture location? Please provide all rupture locations.

**RESPONSE**: As suggested, we have added the sentences in the manuscript to provide all rupture locations.

“Among a total of 20 ruptures found in our study, 2 cases were artery ruptures and the other ruptures occurred at the site where the balloon was inflated. ”

6. Fistula and grafts are different accesses. Please separate these two groups when doing analysis.

**RESPONSE**: We appreciate your valuable suggestion. However, among the 74 VAIVT procedures performed on patients with AVG only, the limited number of outcomes (1) made it difficult to analyze the relationship in patients with AVG. Therefore, we performed a subgroup analysis, focusing only on patients with AVF (excluding those with AVG). The results showed a similar relationship between a high resistance index and an increased rupture risk (as shown in Table 2,4 and Fig2,4). We have included these findings in the manuscript.

“When we reanalyzed the data for patients with AVF only, as a subgroup analysis, the results showed a significant relationship between high resistance index and an increased rupture risk (as demonstrated in Table2,4 and Fig2,4).”

**Table2. Baseline clinical and biochemical characteristics of 553 patients with AVF receiving vascular access intervention therapy. (Wilcoxon-test or chi-square test)**

|  |  | **Not rupture** | **Rupture** | **P-value** |
| --- | --- | --- | --- | --- |
|  |  | N=534 | N=19 |  |
| **Age(year)** |  | 73.00 (64.00-79.00) | 73.50 (68.00-86.00) | 0.28 |
| **sex** | female | 81(15.2%) | 4 (21.1%) | 0.48 |
|  | male | 453 (84.8%) | 165(78.9%) |  |
| **BMI** |  | 22.68(19.91-25.20) | 19.68(18.64-23.55) | 0.038 |
| **Primary disease of ESKD** | Diabetes mellitus | 300(56.3%) | 11 (57.9%) | 0.90 |
|  | Benign nephrosclerosis | 99(18.6%) | 4 (21.1%) |  |
|  | Glomerulonephritis | 81(13.4%) | 1(5.3%) |  |
|  | Polycystic kidney disease | 14 (2.6%) | 0 (0.00%) |  |
|  | Unknown | 49(9.2%) | 3 (15.8%) |  |
| **Hemodialysis vintage(year)** |  | 5.00 (2.00-8.00) | 3.00 (1.00-7.00) | 0.26 |
| **AVF/AVG location** | elbow fistula | 32(6.1%) | 0(0%) | 0.7 |
|  | Radiocephalic fistula | 477(90.3%) | 18(94.7%) |  |
|  | AVF in tabaciere region | 1(0.2%) | 0(0%) |  |
|  | Ulnobasilic fistula | 18(3.4%) | 1(5.3%) |  |
| **Access vintage(year)** |  | 3(1-5) | 1(1-3) | 0.018 |
| **operator** | young | 223(42.9%) | 7(36.8%) | 0.54 |
|  | middle | 277(53.3%) | 12(63.2%) |  |
|  | senior | 20(3.8%) | 0(0%) |  |
| **Stenosis location** | distal anastomose | 255(47.8%) | 5 (26.3%) | 0.066 |
|  | proximal anastomose | 279 (52.2%) | 14 (73.7%) |  |
| **Times of VAIVT** | 1 | 87 (16.3%) | 8 (42.1%) | 0.007 |
|  | 2~4 | 233(43.6%) | 8(42.1%) |  |
|  | 5~ | 214(40.1%) | 3 (15.8%) |  |
| **Qb(ml/min)** |  | 200.00 (180.0-230.0) | 180.00 (150.0-200.0) | 0.005 |
| **FV (ml/min)** |  | 349.0 (238.0-539.0) | 213.0 (179.0-361.0) | 0.006 |
| **RI** |  | 0.63(0.54-0.72) | 0.77(0.62-0.84) | 0.005 |
| **Diameter(before:cm)** |  | 1.00 (0.70-1.30) | 0.85 (0.70-1.10) | 0.23 |
| **Types of balloons** | Non-compliant balloon catheter | 475(89.5%) | 18(94.7%) | 0.46 |
|  | Semi-compliant balloon catheter | 56(10.5%) | 1 (5.3%) |  |
| **sheath size (Fr)** | 4 | 35 (6.6%) | 2 (10.5%) | 0.73 |
|  | 5 | 454(85.2%) | 15(78.9%) |  |
|  | 6 | 44(8.3%) | 2 (10.5%) |  |
| **forwarding/retrograding** | forwarding | 69(13.0%) | 2 (10.5%) | 0.75 |
|  | retrograding | 463 (87.0%) | 17 (89.5%) |  |
| **max inflation atom(atm)** |  | 18.0 (12.0-24.0) | 18.0 (14.0-24.0) | 0.92 |
| **Time of HD(h)** | 3 | 15(2.8%) | 0 (0.00%) | 0.66 |
|  | 3~4 | 473 (88.9%) | 17 (89.4%) |  |
|  | 4~5 | 44 (8.3%) | 2 (10.6%) |  |
| **The amount of removing body fluid(L)** |  | 2.30(1.7-3.0) | 2.0 (1.4-3.0) | 0.33 |
| **CTR (%)** |  | 49.2(45.1-53.1) | 50.2 (48.0-53.8) | 0.22 |
| **WBC(10³/μL)** |  | 5.9(4.8-7.1) | 5.2 (4.5-6.2) | 0.12 |
| **RBC(10⁵/μL)** |  | 349.0(323.0-383.0) | 358.5(329.5-401.5) | 0.39 |
| **MCV (fL)** |  | 97.0(93.3-100.3) | 95.3 (91.2-100.9) | 0.61 |
| **MCH (pg)** |  | 31.2 (29.7-32.6) | 31.5 (28.7-32.2) | 0.62 |
| **Hb(g/dL)** |  | 10.9 (10.3-11.6) | 11.2 (10.3-12.2) | 0.54 |
| **PLT(10⁵/μL)** |  | 18.0(14.4-21.8) | 16.7 (14.6-18.5) | 0.44 |
| **TP(g/dL)** |  | 6.5(6.2-7.0) | 6.50 (6.2-6.7) | 0.57 |
| **Alb(g/dL)** |  | 3.5 (3.3-3.7) | 3.4 (3.2-3.7) | 0.57 |
| **Cre(mg/dL)** |  | 10.2 (8.4-12.4) | 8.7 (7.3-12.0) | 0.14 |
| **BUN (before HD: mg/dL)** |  | 63.0 (53.2-74.2) | 65.0 (56.4-74.6) | 0.68 |
| **BUN (after HD: mg/dL)** |  | 19.7 (15.6-25.0) | 20.2 (17.9-26.3) | 0.52 |
| **URR** |  | 68.6(62.3-72.7) | 67.5 (62.7-71.0) | 0.47 |
| **Calcium(mg/dL)** |  | 8.3 (7.9-8.7) | 8.2 (7.5-8.6) | 0.36 |
| **Phosphorus(mg/dL)** |  | 5.2(4.4-6.1) | 4.9 (4.5-6.3) | 0.84 |
| **iPTH(pg/mL)** |  | 185.5 (121.5-246.0) | 183.0(127.0-308.0) | 0.72 |
| **Fe(μg/dL)** |  | 58.0 (45.0-74.0) | 63.0 (43.0-75.0) | 0.92 |
| **TIBC(μg/dL)** |  | 247.0 (215.0-277.0) | 255.0 (234.0-285.0) | 0.40 |
| **ferritin(ng/mL)** |  | 69.0 (39.0-106.0) | 67.9 (38.5-85.0) | 0.64 |
| **CRP (mg/dL)** |  | 0.17 (0.06-0.47) | 0.20 (0.09-0.43) | 0.83 |
| **Na(mEq/L)** |  | 138.0 (136.0-140.0) | 139.0 (138.0-141.0) | 0.13 |
| **K(mEq/L)** |  | 4.6 (4.1-5.2) | 4.7 (4.4-5.3) | 0.58 |
| **Cl(mEq/L)** |  | 102.0 (99.0-104.0) | 102.00(100.0-105.5) | 0.53 |
| **TSAT (%)** |  | 23.5 (17.8-32.0) | 26.0 (19.0-29.2) | 0.96 |

**Table4. Predictors of rupture based on Multivariate logistic regression analysis for patients with AVF.**

|  | | **OR** | **Lower 95%CI** | **Upper 95%CI** | **p value** |
| --- | --- | --- | --- | --- | --- |
| **age** | | 1.3 | 0.7361999 | 2.463243 | 0.33 |
| **sex** | | 1.1 | 0.2227429 | 5.338613 | 0.92 |
| **Hemodialysis vintage** | | 0.97 | 0.8452117 | 1.109971 | 0.65 |
| **Primary disease of CKD** | | 1.3 | 0.7510335 | 2.100437 | 0.38 |
| **Times of VAIVT** | **First time** | 5.7 | 0.9535718 | 33.92763 | 0.056 |
|  | **More than second** | 2.7 | 0.5287467 | 13.93503 | 0.27 |
| **RI** | | 2.0 | 1.17598 | 3.524161 | 0.011 |
| **Type of balloons** | | 0.50 | 0.0616867 | 4.123893 | 0.523 |
| **Max inflation atom** | | 0.85 | 0.5001578 | 1.445295 | 0.549 |

**Fig2. Relevant to rupture and resistance index for patients (AVF only)**

**Fig 4.ROC curve analysis of resistance index in patients with rupture. (AVF only)**

7. Discussion: “ line 149-150”, “ 20 rupture cases occurred during 627 VAIVT procedures. No ruptures cases occurred after VAIVT in our study”. This sentence is confusing. Can you clarify?

**RESPONSE**: Sorry for the careless errors. We have corrected it.

“All ruptures were diagnosed during VAIVT. No patient was diagnosed after leaving the operation room.”

8. What is the diameter differences of angioplasty balloon and the vessel size? Is the differences between angioplasty balloon and vessel diameter different between rupture group and nonrupture group?

**RESPONSE**: We are grateful for your insightful comments. In our hospital, the operator determined the appropriate size of the balloon based on the diameter of the vessel near the stenosis. We have added the sentence in the manuscript.

“the appropriate size of the balloon was selected by matching slightly oversized in comparison to the caliber of the adjacent normal-appearing vessels.

9. In my opinion, “first time angioplasty” is not a clear variable to predict rupture. For example, fresh fistula has much higher change to rupture than chronic fistula. Please provide detailed information on access vintage. What is the average access age for nonrupture group compared to ruptured group? Please divide into fistula vs graft group. Please provide the time interval between access creation date and procedure date.

→We appreciate your meaningful suggestion. Patients with rupture had significantly shorter access vintage. However, univariate Spearman's Rho correlations did not show any significant association between patients with and without ruptures. Additionally, we undergo VAIVT with a smaller size balloon for immature AVF which might be biased. Therefore, access vintage was not chosen as a variable for the multivariate logistic regression analysis. We have added data in table.

|  | Not rupture | Rupture | P-value |
| --- | --- | --- | --- |
| Access vintage(year) | 3(1-5) | 1(1-3) | 0.018 |

|  | OR | **Lower 95%CI** | **Upper 95%CI** |
| --- | --- | --- | --- |
| Access vintage(year) | 0.8 | 0.6035692 | 1.014585 |

10. Please grade the severity of vessel rupture

**RESPONSE**: We judged that all rupture cases were not so severe because no case needed stent placement, blood transfusion nor emergent surgery.

11. What is the treatment for vessel rupture in your study? what is the success rate?

**RESPONSE**: We have added the text regarding the treatment for vessel rupture.

“All rupture were successfully treated by only balloon tamponade and compression by hand simultaneously at the rupture site. The balloon tamponade was performed at lower pressure for at least 5 minutes but in some cases took time over 15 minutes. “

12. The experiences of proceduralists correlates strongly with the procedure outcome. Please provide detailed information about procedurlists. Are they fellows or attendings? Ir attendings or vascular surgones etc.

**RESPONSE**: In our hospital, all of the doctors operating VAIVT were nephrologists. The proceduralists was below. But, no significant association with rupture. We have added the data in table.

|  |  | **Not rupture** | **Rupture** | **P-value** |
| --- | --- | --- | --- | --- |
| **operator** | fellow | 247(41.6%) | 8(40.0%) | 0.89 |
|  | attending | 347(58.4%) | 12(60.0%) |  |

Reviewer #2: This is a well-written manuscript evaluating in a retrospective fashion the risk factors for vessel rupture during dialysis vascular access angioplasty. The authors described their experience in a single-center setting. While the study does have some limitations, as listed in the discussion (relatively small sample size, single center, avoiding cutting-edge balloons), overall it would be a good addition to the current literature.

I would add to the limitations {low Qb of 200 ml/min and vascular access flow volume (180-540 ml/min)}, which perhaps is the standard in Japan, but considered low in other countries.

We appreciate your comments making our paper more solid. We agreed with it and have added the sentences in the manuscript.

“Fourth, Qb of 200 ml/min and vascular access flow volume (180-540 ml/min) are common in Japan, but which relatively smaller than those in the western country”

Thank you for evaluating our manuscript and providing us with valuable comments, and we hope that you agree that the changes made have markedly improved our manuscript.

We hope our revised manuscript will be accepted for publication in *PLOS ONE*.

Thanking you in anticipation,

Sincerely,

Correspondence to: Yui Kambayshi , M.D.

Division of Nephrology, Kanto Rosai Hospital, 1-1 Kizukisumiyoshicyou, Nakahara-ku, Kawasakishi, Kanagawaken, Japan

E-mail; [hanamay33@outlook.jp](mailto:hanamay33@outlook.jp)
